# Photonic topological fermi nodal disk in non-Hermitian magnetic plasma

**DOI:** 10.1038/s41377-020-0274-3

**Published:** 2020-03-11

**Authors:** Wenhui Wang, Wenlong Gao, Leifeng Cao, Yuanjiang Xiang, Shuang Zhang

**Affiliations:** 1 International Collaborative Laboratory of 2D Materials for Optoelectronic Science & Technology of Ministry of Education, Institute of Microscale Optoelectronics (IMO), Shenzhen University, 518060 Shenzhen, China; 2 School of Physics & Astronomy, University of Birmingham, Birmingham, B15 2TT UK; 3 National Key Laboratory for Laser Fusion, Research Centre of Laser Fusion, CAEP, 621900 Mianyang, China; 4 School of Physics and Electronics, Hunan University, 410082 Changsha, China

**Keywords:** Sub-wavelength optics, Magnetically confined plasmas

## Abstract

Topological physics mainly arises as a necessary link between properties of the bulk and the appearance of surface states, and has led to successful discoveries of novel topological surface states in Chern insulators, topological insulators, and topological Fermi arcs in Weyl, Dirac, and Nodal line semimetals owing to their nontrivial bulk topology. In particular, topological phases in non-Hermitian systems have attracted growing interests in recent years. In this work, we predict the emergence of the topologically stable nodal disks where the real part of the eigen frequency is degenerate between two bands in non-ideal magnetohydrodynamics plasma with collision and viscosity dissipations. Each nodal disk possesses continuously distributed topological surface charge density that integrates to unity. It is found that the lossy Fermi arcs at the interface connect to the middle of the projection of the nodal disks. We further show that the emergence, coalescence, and annihilation of the nodal disks can be controlled by plasma parameters and dissipation terms. Our findings contribute to understanding of the linear theory of bulk and surface wave dispersions of non-ideal warm magnetic plasmas from the perspective of topological physics.

## Topological band theory helps explain warm plasma dynamics

Scientists have applied a modern theory to gain further insight into the century-long study of warm plasma, a product of nuclear fission reactions. ‘Topological band theory’ was recently developed to describe electron energies in a type of material that is insulating on its inside and conducting on its surface. Shuang Zhang of the University of Birmingham, UK, and colleagues applied the theory to further understand electromagnetic wave dynamics in warm plasma, a gas consisting of freely moving ions, nuclei and electrons. The team’s equations incorporated information on dissipations from particle collisions and viscosity forces, allowing them to predict some of the nontrivial topological behaviors within warm plasma. Their work is of benefit to fundamental science and to improving understandings of the solar corona, synchrotron radiation, and the production of energy from controlled thermonuclear reactions.

## Introduction

Plasma, widely considered as the fourth states of matter, is a completely ionized gas consisting of freely moving ions, nuclei, and electrons. Plasma is believed to account for over 99% of the matters in the universe. Understanding the bulk and the surface wave dispersions of plasma’s magnetic hydrodynamics is of great importance for both fundamental interests in topological matter states and broad applications in fusion energy harvesting, plasma diagnostics etc. In applications with low particle density and negligible temperature effect such as neon tubes^1^, known as “cold plasma”, the charged particles are assumed to oscillate without significant inter-particle interactions due to the diminishing Lamor radius^2^. Landau^3^ considered magnetic fluids as continuous media and used the cold plasma theory to describe the excitations in plasma as frequency dependent, local (meaning no spatial dispersion) dielectric functions. Such electrodynamics of continuous media theory has been experimentally testified to give satisfying descriptions of zonal flows, Geodesic acoustic mode, and Alfven waves^4^.

However, in higher energy regimes, such as thermonuclear weapons, tokamak configuration with magnetic and inertial confinement, and intense X-ray sources, the particle density is immensely higher, and the temperature effect cannot be neglected^5–7^. As a consequence, the dynamics of the plasma are thermal-dependant and described by the Navier–Stokes equations, and are known as “warm plasma”, whose dielectric functions are not only frequency dependant but also spatially dispersive (nonlocal). Study of the bulk and surface wave dispersions of the “warm plasma” can be used for plasma diagnostics and for understanding astrophysical problems in the magnetosphere, the solar corona and surface waves enhanced electron heating, ion acceleration, synchrotron radiation, and static magnetic generation. Furthermore, surface waves are also involved in laser fusion, which is valuable for energy productions through controlled thermonuclear reactions (CTR)^8–10^.

It has been shown previously that Weyl points may exist in cold plasma, leading to observation of interesting topological effects such as presence of Fermi arcs^11,12^. Since large dissipation terms could arise in warm plasma^13,14^, they provide an interesting platform for studying non-Hermitian topological physics, which has become a vibrant research field in recent years^15–25^. Topologically protected degeneracies in Hermitian systems such as Weyl points are stable, due to the participation of all three Pauli matrices in constructing the low-energy Hamiltonian. However, with additional degree of freedom from the loss/gain, the probability of finding a degeneracy is lower than is dictated by the Von Neumann–Wigner theorem^26^. This means the two band Hamiltonian may experience level repulsion lifting the degeneracies. For instance, the conical refraction phenomenon could be destroyed by introducing another “loss optical axis”^27^. However, interestingly, rather than simply lifting the degeneracy, the non-Hermicity may turn a Weyl point into a two-dimensional nodal disk where the real part of the complex eigen frequencies becomes degenerate, which is surrounded by an exceptional loop (EL), where both the complex eigen frequency and eigen-states coalesce. Till now, besides being theoretically investigated in a minimum 2-by-2 Hamiltonian model^15,28–31^, there have been only a few studies on topological ELs in optical lattices and woodpile photonic crystals^32–34^.

In this work, we predict the emergence of topologically stable nodal disks and ELs in non-ideal magnetohydrodynamics (MHD) plasma by taking into account the dissipations, including collisions and viscosity forces. While previous works have focused on ELs, we find the nodal disks to be more topologically important than the ELs mainly in two aspects: (1). The Berry curvatures are primarily emitted from the nodal disks. (2). The Fermi arcs connect to the centers of the nodal disks projected onto the interface. These nodal disks are non-Hermitian generalizations of the Weyl points whose topological charge can be confirmed by the distribution of non-Hermitian Berry curvature under bi-orthogonal basis^15,35–38^. Momentum-space positions of nodal disks are sensitive to the plasma temperature, magnitude of the magnetic field, and the viscosity force. They could coalesce and annihilate through variation of these parameters, and this phenomenon has not been readily found in other systems. In the lossless limit, these nodal disks reduce into type-II Weyl points and could further transform into transitional Weyl points, which lies between type-I and type-II with the dispersion of one participating mode being perfectly flat, by reducing the plasma temperature to zero. Dissipative surface states (Fermi arcs) are also found to emanate from the topologically charged nodal disks. Interestingly, viscosity force is found to introduce extra type-I nodal disks at larger momentum. By tuning the magnitude of the viscosity loss, the extra type-I nodal disks could coalesce and annihilate with type-II nodal disks. Our study introduces the modern topological band theory into the century-long research field of warm plasma.

## Results

### Hamiltonian formalism derived from the plasma fluid dynamics

In a warm plasma, the charged particles’ macroscopic motion can be described by the fluid dynamics (Navier–Stokes equation) with parameters, such as pressure and density. In contrast to conventional fluid where only longitudinal wave exists due to the absence of tangential resilience, warm plasma’s dispersion is more complicated due to the presence of electromagnetic force. In this work, MHD in combination with Maxwell equations are used to describe the fluid motions of the plasma under electromagnetic interactions. Taking into account the dissipation terms, which include the collision and viscosity, the plasma fluid dynamics equation is given as,

1 $$\frac{{\partial {\boldsymbol{u}}_{\boldsymbol{e}}}}{{\partial t}} + {\boldsymbol{u}}_{\boldsymbol{e}} \cdot \nabla {\boldsymbol{u}}_{\boldsymbol{e}} = q_en_{\mathrm{e}}\left( {{\boldsymbol{E}} + \frac{1}{c}{\boldsymbol{u}}_{\boldsymbol{e}} \times {\boldsymbol{B}}} \right) - \frac{{\nabla P_{\mathrm{e}}}}{{{\it{n}}_{\it{e}}{\it{m}}_{\it{{\mathrm{e}}}}}} + \frac{{{\boldsymbol{u}}_{\boldsymbol{e}}}}{\tau } + \eta \nabla ^2{\boldsymbol{u}}_{\boldsymbol{e}}$$

Where *n*_*e*_ is the plasma density, *m*_e_ is the electron mass, *q*_e_ is the elementary charge, *P*_e_ is the electrons thermal pressure, *η* is the viscosity coefficient, which arises from the frictions between counter-propagating electrons, *τ* is the mean free time between electrons collision. The corresponding Hamiltonian formalism is given as (Supplementary Note 1 for detail).

2 $$\omega _p\left[ {\begin{array}{*{20}{c}} {} & { - {\boldsymbol{K}} \times /k_P} & {} & { - i} \\ {{\boldsymbol{K}} \times /k_P} & {} & {} & {} \\ {} & {} & {} & {\kappa {\boldsymbol{K}}/k_P} \\ i & {} & {\kappa {\boldsymbol{K}}/k_P} & {\omega _c\Delta - iI/\tau - iI\eta K^2} \end{array}} \right]\left[ {\begin{array}{*{20}{c}} {\boldsymbol{E}} \\ {\boldsymbol{H}} \\ n \\ {\boldsymbol{j}} \end{array}} \right] = \omega \left[ {\begin{array}{*{20}{c}} {\boldsymbol{E}} \\ {\boldsymbol{H}} \\ n \\ {\boldsymbol{j}} \end{array}} \right]$$

where $\omega _p = \left( {n_0e^2/m_e} \right)^{1/2}$ is the equilibrium plasma frequency, $\omega _c = \frac{{q_eB}}{{m_ec}}$ is the electron cyclotron frequency, $k_p = \omega _p/c$, Δ = $\left[ {\sigma _y\,0;0\,0} \right]$, *I* is the identity matrix, and ${\upkappa}^2 = \frac{{P_0\gamma }}{{n_0mc^2}}$ with *γ* being the ratio of specific heats, *n* is the fluctuation of plasma density, *j* the polarized current. In the rest of the paper, the frequencies are normalized by *ω*_*p*_, *η* has unit of ${\mathrm{kg}}/\left( {{\mathrm{m}} \!\cdot\! {\mathrm{s}}^2} \right)$ corresponding to actual viscosity coefficient to *η*/*ω*_*p*_ whose unit is ${\mathrm{kg}}/\left( {{\mathrm{m}} \!\cdot\! {\mathrm{s}}} \right)$. The traditional mathmatically complicated calculation of the nonlocal dielectric functions is thus transformed into an eigen-value problem.

**Fig. 1:**
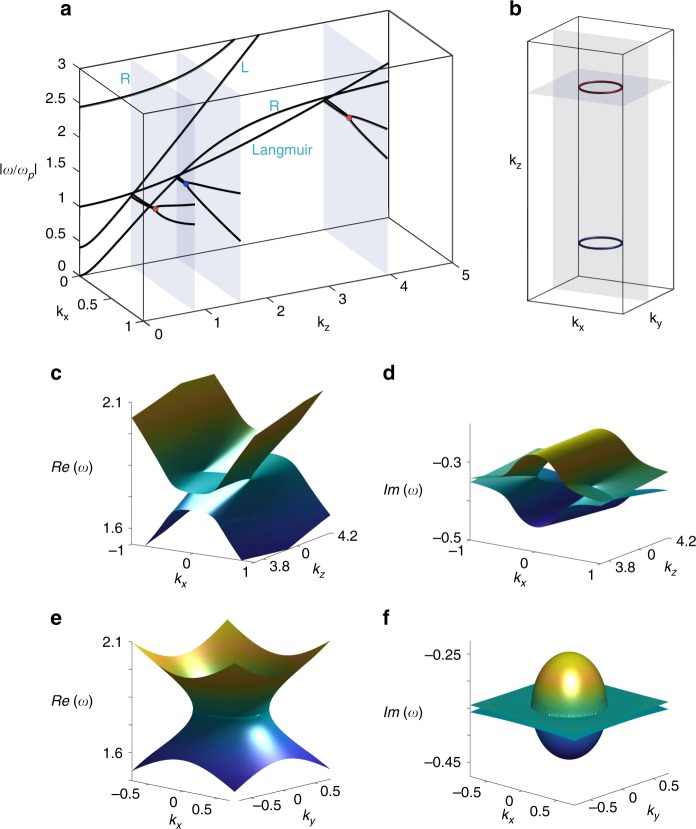
Existence of Nodal disks in magnetohydrodynamic plasma. **a** Dispersion of the absolute value of eigen frequency *ω* with $\omega _c = 2,\,\kappa = 0.4,\,\tau = 2$. Along the *k*_*z*_ axis, the bands are differentiated as right/left handedness circular polarization wave (R/L-wave) and Langmuir wave whose polarization is only in the *z*-direction. The bands along transverse directions (*x*-direction) have degeneracies denoted by the red/blue dots. Owing to the rotational symmetry in the plasma system, the dots in **a** form the loops in **b**. Akin to the Weyl points, there are two species of Nodal disks in the plasma system with opposite topological charge (+/–1 charge denoted by the red/blue color) that are connect by the spatial inversion operation. **c**–**f** Real and imaginary part of the eigen frequency in the *k*_*x*_−*k*_*z*_ and *k*_*x*_−*k*_*y*_ plane. Note that in the *k*_*x*_−*k*_*z*_ plane, real and imaginary parts are simultaneously degenerated at isolated points away from the *k*_*z*_ axis. While in the *k*_*x*_−*k*_*y*_ plane, the degeneracies form a ring

We start by considering only the collision dissipation while neglecting the effect of viscosity. The dispersion of MHD plasma can be solved by diagonalizing the Hamiltonian in Eq. (1). The absolute value of the complex frequency of the non-ideal magnetized warm plasma is shown in Fig. 1a with $\omega _p = 3.5 \times 10^{12}\,{\mathrm{rad}}/{\mathrm{s}}$, $\omega _c = 2\omega _p$, *κ* = 0.4, *τ* = 2, corresponding to a magnetized plasma system with an electron density of $3.9 \times 10^{19}\,1/{\mathrm{m}}^3$, an external magnetic field B of 8.01 T, an electron thermal temperature of 27.3 keV and an electronic collision frequency of $1.75 \times 10^{12}\,{\mathrm{rad}}/{\mathrm{s}}$. The band structure shows four bands, which, ordered from low to high frequencies, are the first right-handed circularly polarized band (R-wave), the longitudinal Langmuir (LM) wave (see detail in Supplementary Fig. 1), the left-handed circularly polarized band (L-wave) and the second band of R-wave. In the lossless limit R-wave and L-wave’s dispersions are expressed as $k_{{\mathrm{R/L}}} = \frac{\omega }{c}\left[ {1 - \frac{{\omega _{pe}^2}}{{\omega ^2\left( {1 \mp \omega _{ce}/\omega } \right)}}} \right]^{1/2}$. The LM wave’s dispersion is given by $\omega ^2 = \omega _{pe}^2 + \kappa ^2c^2k^2$, which can reduce to the cold plasma’s^4^ oscillation frequency by taking *κ* = 0. (Supplementary Note 2 for detail and Supplementary Fig. 2). The positions of the Weyl points are caculated in Supplementary Fig. 3. To better understand these linear band crossing, we apply the *k·p* theory (effective Hamiltonian theory) under collisionless conditions to obtain the approximate Hamiltonian near the degeneracies. Expanding to first order in the vicinity of the outer degeneracy, we find the effective Hamiltonian as:

3 $$H_1 = \frac{{A + N}}{2}k_z\sigma _z + Mk_x\sigma _x - Mk_y\sigma _y + \frac{{A - N}}{2}Ik_z$$

where $A = \frac{{\sqrt {\varepsilon _{12}} }}{{2\left( {\varepsilon _{12}^2\, -\, \varepsilon _{12} \,+\, 2} \right)}}$, $N = \frac{{\omega _p^2\sqrt {\omega _c} }}{{2\left( {\omega _p \,+\, \omega _c} \right)\sqrt {\omega _c \,-\, \omega _p} }}\kappa ^2$, $M = \frac{{\sqrt {\varepsilon _{12}} }}{{2\sqrt {\varepsilon _{12}^2 \,-\, \varepsilon _{12} \,+\, 2} }}$, and *ε*_12_ is sum of the diagonal and off-diagonal elements of the dielectric matrix evaluated at the Weyl’s frequency, which can be expresses as $\omega _{{\mathrm{weyl}}} = \omega _p + \frac{{\omega _p^2\omega _c}}{{2\left( {\omega _c^2 \,-\, \omega _p^2} \right)}}\kappa ^2$ (Supplementary Note 3 for detail). These linear degeneracies are Weyl points that function as the sources and drains of Berry curvature flux lines. Owing to the electrons collision, the new degeneracy points where both the real and the imaginary part of the complex frequency spectrum are degenerate shift away from the *k*_*z*_ axis, as denoted by the colored dots in Fig. 1a. Owing to the rotational symmetry of the system around *k*_*z*_ axis, the degeneracy points form exceptional loops in the *k*_*x*_-*k*_*y*_ plane in Fig. 1b. Figure 1c, d show the real and imaginary parts of the eigen frequency in *k*_*x*_–*k*_*z*_ direction, where the degeneracy line for the real part of the eigen frequency in the *k*_*x*_–*k*_*z*_ plane forms a bulk nodal disk due to the rotational symmetry. The dispersion of the real and imaginary parts of the eigen frequency on the *k*_*x*_-*k*_*y*_ plane are presented in Fig. 1e, f, respectively, which show the presence of an EL. As shown in Fig. 1, the modified dispersion of the LM wave is curved due to the thermal effect, providing the possibility of forming three pairs of nodal disks between the LM Wave and the L/R-wave. In contrast the cold plasma can at most possess two pairs of Weyl points due to its dispersionless LM mode^39^. As shown in Fig. 1a, two types of band-crossing exist: the band crossings between the LM wave and the L-wave at small *k*, and the other two between the LM wave and the first R-wave at larger *k*’s. These band crossings are guaranteed by the polarization orthogonality between the longitudinal LM waves and the transverse L and R waves.

### Topological phase transition in the dissipating collision plasmas

**Fig. 2:**
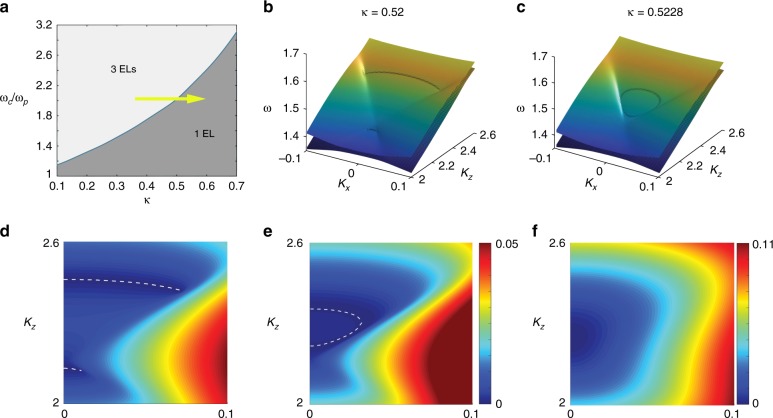
Phase map, the emergence, and annihilations of the exceptional loops at τ = 2. **a** The number of nodal disks is due to change by the strength of magnetic field *ω*_c_ and the electron thermal parameter *κ*. Along the path crossing the border line, for example the path shown as the yellow arrow in **a**, the annihilation or emergence of nodal disks could be observed. Three-dimensional (3-D) band structures before and at the critical transition point are given in **b** and **c**. Difference of the real part of the eigen frequencies around the nodal disks before, at and after the annihilation of the nodal disks are given in **d**–**f**. The dashed lines represent the locations where the difference goes to zero. The positions of the nodal disks are located at the edge of the dashed lines. **d** Before the coalesce of the two nodal disks. **e** At the border between the two phases, the two nodal disks would coalesce and dissipate their topological charges. **f** After the coalesce, the zeros in the **e** would shift from zero

By tuning the thermal parameter *κ* and the cyclotron frequency *ω*_c_ under a fixed collision coefficient *τ* = 2, the nodal disks’ location in the momentum space can be shifted along the *k*_*z*_ direction, or even merged or annihilated. In Fig. 2a, the transition of the number of nodal disks is illustrated in a phase map in the parameter space (*ω*_c_, *κ*), which shows that the system acquires either three pairs or a single pair of nodal disks. Right at the phase boundary, the two nodal disks at larger *k*_*z*_ join with each other. The difference in the real part of the frequency between the LM wave and R-wave constituting the nodal disks for different *κ* values is given in Fig. 2d–f. As shown in Fig. 2d, when *κ* = 0.52, *ω*_c_ = 2 (below the critical transition value), the nodal disks are separate (white dash lines). While at the critical transition value *κ* = 0.5228, *ω*_c_ = 2 the exceptional loops merge into each other and the nodal disks form a closed nodal surface, as shown in Fig. 2e. The corresponding 3-D band structures to Fig. 2d, e are given in Fig. 2b, c. It should be noted that even though the Chern number of the closed nodal surface is zero since the topological charges of the two nodal disks cancel each other, the Berry curvatures do not vanish because of the nonzero topological charge density on the surface. Further increase of *κ* beyond the critical value leads to simply shrinkage of the closed nodal surface and lifting of the degeneracy as is shown in Fig. 2f.

### Berry curvatures and topological dissipating fermi arc of the bulk nodal disk

**Fig. 3:**
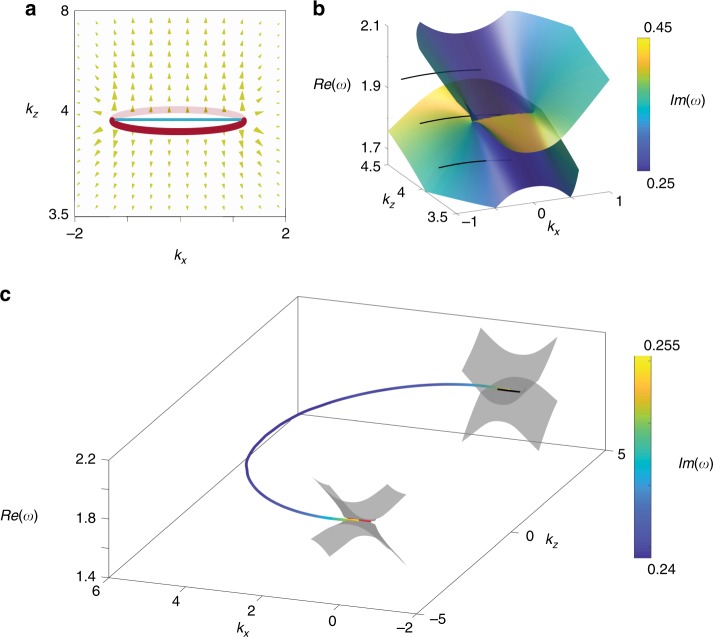
Berry curvature distribution and dissipative fermi arcs of the nodal disks with $\omega _c = 2,\,\kappa = 0.4,\,\tau = 2$. **a** Berry curvature for the upper band of collision warm magnetic plasma in the *k*_*x*_−*k*_*z*_ plane with. The red rings are the surrounding of the Nodal disks in *k*_*x*_−*k*_*y*_ plane and the blue solid line is the projection of the bulk nodal disk in *k*_*x*_−*k*_*z*_ plane. Scales of the arrow represent the intensity of Berry curvatures and orientation indicates the direction. The arrows show that the bulk nodal disks are pure source of Berry curvature. **b** fermi arcs connect at the larger (1.96), equals (1.86), and smaller (1.76) bulk nodal disk frequency. For the Nodal disks frequency, Fermi arc connects at center of the real dependency line while Fermi arc emits from the bulk states when the frequency shifts. The color represents the value of the corresponding imaginary frequency. **c** dissipating fermi arc between plasma and vacuum, which connects the two bulk nodal disk with opposite chirality, the color represents the value of the corresponding imaginary frequency

Similar to Weyl points in Hermitian systems, nodal disks are sources and drains of Berry curvatures in non-Hermitian systems. In such a dissipative system, Berry curvatures can be derived from any entry of the Berry connection matrix: $A_\mu ^{I,J} = i\langle I{\mathrm{|}}\partial _\mu {\mathrm{|}}J\rangle,\,I,J \in \left\{ {L,R} \right\}$. In Supplementary Note 5, it is shown that this matrix is Hermitian, whose diagonal entries are real, and off-diagonal complex. The matrix entries satisfy the relation: $A_\mu ^{L,L} = A_\mu ^{R,R}\, \ne\, A_\mu ^{L,R} = A_\mu ^{L,R \ast }$, where the sign * stands for the complex conjugate. It could be further shown that the Berry curvature calculated from different matrix entries are locally different, however, their integral over the nodal disk, the Chern numbers, are the same (for the complex term $A_\mu ^{L,R}$ and $A_\mu ^{L,R}$, only real parts are integrated)^15^. In dissipative MHD plasma, Berry curvature flux distribution from the nodal disk between the LM and the R waves is shown in Fig. 3a, where the red rings represent the ELs in *k*_*x*_−*k*_*y*_ plane and the blue solid line is the projection of the nodal disk in *k*_*x*_−*k*_*z*_ plane. Figure 3a clearly shows that the nodal disk is the source of Berry curvature. In proximity to the EL, the derived Berry curvatures diverage is at the rate of $1/\!\sqrt r$, where *r* is the distance to the exceptional loop (see detail in Supplementary Note 5). This means the Berry curvature emitted by the exceptional loop has neligible value. Hence it is confirmed that the topological charge is continuously distributed across the bulk nodal disks, rather than concentrated at the ELs.

Owing to the nontrivial topological charge of the nodal disk, presence of Fermi arcs is expected. To solve for the Fermi arc, we consider an abrupt interface between the MHD plasma and vacuum, which can be found in magnetic confinement plasmas such as Tokamak configurations. Because of the nonlocality of the system, an additional boundary condition that the current density diminishes in the surface normal direction should be imposed, together with the conventional electromagnetic wave continuity conditions. Considering the continuity of bulk displacement current normal to the interface, this additional boundary condition is equivalent to continuity of *E*_x_ across the interface^40^. The dissipating Fermi arc near the outer the nodal disk at the real frequency parts of 1.76 *ω*_p_, 1.86 *ω*_p_, and 1.96 *ω*_p_ is given in Fig. 3b (more details are shown in Supplementary Fig. S7). Interestingly, at the EL frequency, the dissipative Fermi arc connects between the projected nodal disks right in the middle, as shown in Fig. 3c. However, away from the exception loop frequency, the Fermi arc could exist inside the bulk state continuum, as shown in Fig. 3b. Detailed derivations are provided in the Supplementary Note 4, and the surface modes at different frequencies are presented in Supplementary Fig. S4.

### Extra nodal disks and topological transition induced by viscosity force

**Fig. 4:**
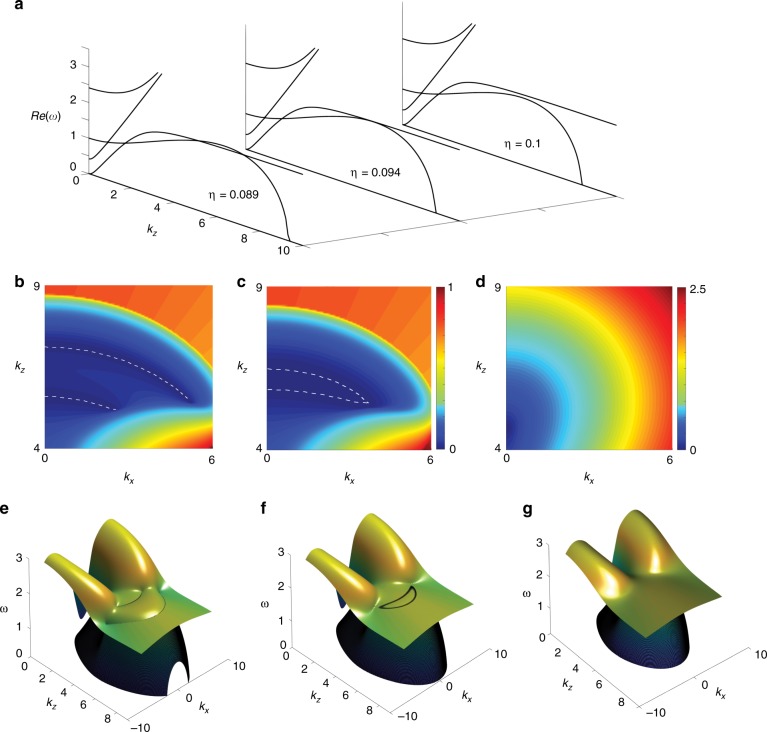
Viscosity force induced extra Nodal disks and their phase transitions. **a** According to different viscosity parameter range at $\omega _c = 2,\,\kappa = 0.4$, the magnetohydrodynamic plasma could have either four pairs of Nodal disks or two pairs by the annihilations of the 2nd and the 3rd, or the 3rd and the 4th. White dash lines represent the real parts’ degeneracy line in the momentum plane of *x*–*y*. Difference of the real part of the eigen frequencies **b** before, **c** at, and **d** after near to the exceptional loops’ annihilation by increasing the viscosity force. The 3-D band structures are give in **e**–**g**

In the above, we have studied the warm magnetic plasma with collision loss. However, in tokamaks and helical devices, there also exists strong anomalous viscosity^14^. As indicated by Eq. (2), viscosity leads to an extra quadratic term in the dispersion of the LM wave, which becomes significant at large momentum. The significantly modified dispersion of the LM wave could introduce an extra pair of nodal disks by intersecting the R-wave again. The real part of the eigen frequency spectrum around the nodal disks is given in Fig. 4a. Comparing with that in a plasma with only collisions, the nodal disk in a plasma with viscosity dissipation is highly curved due to the presence of the higher order term in the momentum. The curved nodal disks can be attributed to the $k_{x,y}^2$ terms present in the effective Hamiltonian model (Supplementary Note 7). Increasing the strength of the viscosity force could induce coalescence and annihilation of the nodal disks as shown in Fig. 4b–d. Similar to the effects shown in previous sections, at a certain viscosity force, tuning the magnetic field strength and electron thermal parameter, the 2nd and 3rd nodal disks could annihilate, leaving only the 1^st^ and the 4th exceptional loops.

## Discussion

In this paper, we have studied the nodal disks and the topological band theory in MHD plasma by considering the collision and viscosity effects. Electron thermal pressures drives the plasma oscillation (longitude mode) propagation and modifies the dispersion slope. Topological charged nodal disks are observed due to the dissipation effects arising from collision and viscosity. Topological protected dissipating fermi arcs are observed, which connects to the bulk nodal disk. Importantly, the emerge, coalesce, and annihilation of these nodal disks can be tuned by thermal parameter and the external magnetic field. Our discoveries could facilitate studies of both the linear theory of MHD plasma and the non-Hermitian topological band theories.

## Methods

Plasma encompass a very large range of scales in length, density and temperature. Our theoretically predicted topological phase transitions could be observed in a broad range of the above parameters. For simplicity we normalize the cyclotron frequency *ω*_c_, the electron collision frequency 1/*τ* and the viscosity coefficient *η* by the plasma oscillation frequency *ω*_p_. In our work, the electron density is $3.9 \times 10^{19}\,1/{\mathrm{m}}^3$, which corresponds to the plasma frequency $\omega _{\mathrm{p}} = 3.5 \times 10^{12}\,{\mathrm{rad}}/{\mathrm{s}}$, the electron thermal temperature of 27.3 keV with an external magnetic field of 8.01 T.

The dissipative surface states in our work are obtained by two methods: (1) solving the plane wave solution of Eq. (2) followed by matching the boundary condition and (2) numerical simulation with COMSOL, a commercially available software. In the first method, extra boundary condition demanding the continuity of the surface normal direction’s electric field is added. This extra condition ensures the plasma will not spill out into the surrounding medium. In COMSOL, the surface states are solved using the generalized PDE module, and the results can be found in Supplementary Fig. S4 and Supplementary Note 6 in the Supplementary Information.

## Supplementary information

Supplementary Material for Photonic topological fermi nodal disk in non-Hermitian magnetic plasma
