# Supplementary material for: Photonic topological fermi nodal disk in non-Hermitian magnetic plasma
Source: Light Sci Appl. 2020 Mar 11;9:40. doi: 10.1038/s41377-020-0274-3 (PMC7066160; doi:10.1038/s41377-020-0274-3)
Supplement: Supplementary file 1 — Supplementary Material for Photonic topological fermi nodal disk in non-Hermitian magnetic plasma [file 41377_2020_274_MOESM1_ESM.docx]

**Supplementary Information for：**

**Photonic topological fermi nodal disk in non-Hermitian magnetic plasma**

Wenhui Wang^1, 2*^, Wenlong Gao^2*^, Leifeng Cao^3^, Yuanjiang Xiang^4^, Shuang Zhang^2^

1. International Collaborative Laboratory of 2D Materials for Optoelectronic Science & Technology of Ministry of Education, Institute of Microscale Optoelectronics (IMO), Shenzhen University, Shenzhen 518060, China
2. School of Physics & Astronomy, University of Birmingham, Birmingham, B15 2TT, UK
3. National Key Laboratory for Laser Fusion, Research Center of Laser Fusion, CAEP, Mianyang 621900, China

4. School of Physics and Electronics, Hunan University, Changsha, 410082, China

Correspondence:Yuanjiang Xiang (xiangyuanjiang@126.com); Shuang Zhang(s.zhang@bham.ac.uk)

^*^ These authors contribute equally

Note 1*Hamiltonian*:

To consider the possible modes in a plasma, here we always assume an equilibrium inthe plasma which parameters such as density and magnetic are uniform and constant in time. And we take a small perturbation away from equilibrium to study time and space dependence modes due to these perturbations, where$n_{1}$and$v_{1}$shows$e^{j\left( \vec{k}\cdot\vec{r}-\omega t \right)}$variation. Here we use the subscript 0 to label equilibrium parameters and for the perturbed with a subscript 1. We assume that the perturbations are much smaller as $|\frac{n_{1}}{n_{0}}|\ll1$. We can transform the plasma states Equation into formalism $dP_{e}n_{e}^{-\gamma}/dt=0$,which gives $P_{1}=\gamma n_{1}P_{0}/n_{0}$. We generally ignore squares and higher order terms, thus we could obtain a set of equations by substitutingsup equation (1) into the main text equation (1).

$\begin{matrix} \omega n_{e}=n_{0}\vec{k}\cdot\vec{u}_{e} \\ \omega\vec{u}_{e}=i\frac{e}{m}(\vec{E}+\frac{\vec{u}_{e}\times\vec{B}_{0}}{c})+\frac{\gamma\vec{k}P_{0}n_{e}}{n_{0}^{2}m} \end{matrix}$ (1)

Combing these equations above and Maxwell equations, extent these equations in each component.

$\begin{matrix} \omega E_{x}=-c(k_{y}H_{z}-k_{z}H_{y})-ij_{x} \omega H_{x}=c(k_{y}E_{z}-k_{z}E_{y}) \\ \omega E_{y}=-c(k_{z}H_{x}-k_{x}H_{z})-ij_{y} \omega H_{y}=c(k_{z}E_{x}-k_{x}E_{z}) \\ \omega E_{y}=-c(k_{x}H_{y}-k_{y}H_{x})-ij_{z} \omega H_{z}=c(k_{x}E_{y}-k_{y}E_{x}) \\ \omega n_{e}=n_{0}\vec{k}_{x}\cdot\vec{u}_{ex}+n_{0}\vec{k}_{y}\cdot\vec{u}_{ey}+n_{0}\vec{k}_{z}\cdot\vec{u}_{ez} \\ \omega j_{x}=i\frac{e^{2}n_{0}}{m}E_{x}+\frac{(j_{y})B_{0}}{cm}+\frac{\gamma k_{x}P_{0}n_{e}^{'}}{n_{0}^{2}m}k_{p} \\ \omega j_{y}=i\frac{e^{2}n_{0}}{m}E_{y}+\frac{(-j_{x})B_{0}}{cm}+\frac{\gamma k_{y}P_{0}n_{e}^{'}}{n_{0}^{2}m}k_{p} \\ \omega j_{z}=i\frac{e^{2}n_{0}}{m}E_{z}+0+\frac{\gamma k_{y}P_{0}n_{e}^{'}}{n_{0}^{2}m}k_{p} \end{matrix}$(2)

By taking the collision term into account, the derivate Hamiltonian formalism of the warm magnetic plasma as below:

$\omega_{p}\left[\begin{matrix} & -K\times/k_{P} & & -i \\ K\times/k_{P} & & & \\ & & & \kappa K/k_{P} \\ i & & \kappa K/k_{P} & \omega_{c}\Delta-i\tau I \end{matrix}\right]\left[\begin{matrix} E \\ H \\ n \\ j \end{matrix}\right]=\omega\left[\begin{matrix} E \\ H \\ n \\ j \end{matrix}\right]$(3)

Note 2

*longitude mode:*

plasma as the qusi-equilibrium states, electrons were pushedfrom equilibrium positions and then pulled back due to electrostatic force which is $k\cdot E_{1}+en_{e1}/\varepsilon_{0}=0$. Combing with the plasma equations (1-3) in the main text, we can obtain the frequency of plasma

oscillation$\omega_{p}=\left( n_{0}e^{2}/m_{e} \right)^{1/2}$which cannot propagate. However when the [electron](https://en.wikipedia.org/wiki/Electron) thermal effects are taken into account, the electron pressure acts as a restoring force as well as the electric field and the oscillations propagate with frequency and [wavenumber](https://en.wikipedia.org/wiki/Wavenumber) related by the longitudinal Langmuirwave which is shown in FIG. S1:

$\omega^{2}=\omega_{pe}^{2}+c^{2}\kappa^{2}k^{2}$(4)

While for the cold magnetic plasma,$\kappa=0$,longitude mode describes oscillations, not waves, which is$\omega^{2}=\omega_{pe}^{2}$. This is plasma oscillations, and the plasma oscillation frequency is just the plasma frequency.

*Electromagnetic waves(R and L waves)in plasma:*

Transvers waves travel along z axis, which have in plane electric field. And the magnetic force affects the electron movements which are still transverse. Here we take the $k_{z}$components and list each component of the main text equation (1), eliminate$\vec{B_{1}}$ and$\vec{j_{1}}$, leaving equations in $\vec{E_{1}}$

$$\left\{\begin{matrix} -i\omega m_{e}u_{x}=-eE_{1x}-eu_{y}B_{0} 5(a) \\ -i\omega m_{e}u_{y}=-eE_{1y}+eu_{x}B_{0} 5(b) \\ (\omega^{2}-k^{2}c^{2})E_{1x}=ie\omega n_{0}u_{x}/\varepsilon_{0} 5(c) \\ (\omega^{2}-k^{2}c^{2})E_{1y}=ie\omega n_{0}u_{y}/\varepsilon_{0} 5(d) \end{matrix}\right.$$

Sub 5(c)and 5(d) into (1) and (2) obtains,

Obtains the dispersion of the electromagnetic waves,

$\omega^{2}-k^{2}c^{2}-\omega_{pe}^{2}=\pm\frac{\omega_{ce}}{\omega}(\omega^{2}-k^{2}c^{2})$ (7)

Sub equation (7) to equation (6), obtains $E_{1y}=\pm iE_{1x}$, which means the Electromagnetic waves along the External magnetic field are circle polarized. And the dispersion relationship can be obtained by the form as,

$k_{R/L}=\frac{\omega}{c}\left[1-\frac{\omega_{pe}^{2}}{\omega^{2}(1\mp\omega_{ce}/\omega)}\right]^{1/2}$ (8)

which$k_{R/L}$ is the wave vector of the vector of right/left handed waves.

Note 3:

*Effective Hamiltonian*

The spirit of the approximation method, is to expand the eigenstates at$\vec{k}=\vec{k}_{0}+\delta\vec{k}$on the basis of $\vec{k}_{0}$which is the Weyl points’ coordinate in momentum space. Take the eigenvalues and eigenvectors at$\vec{k}_{0}$as a pre-condition, we have:$H(\vec{k}_{0})|n(\vec{k}_{0})\rangle=\omega_{n}(\vec{k}_{0})|n(\vec{k}_{0})\rangle$.

And at $\vec{k}=\vec{k}_{0}+\delta\vec{k}$we have $H(\vec{k}_{0}+\delta\vec{k})|n(\vec{k}_{0}+\delta\vec{k})\rangle=\omega_{n}(\vec{k}_{0}+\delta\vec{k})|n(\vec{k}_{0}+\delta\vec{k})\rangle$

Expand$|n(\vec{k}_{0}+\delta\vec{k})\rangle$ on$|n(\vec{k}_{0})\rangle$, we have: $|n(\vec{k}_{0}+\delta\vec{k})\rangle=\sum_{J} |\mathrm{J}(\vec{k}_{0})\rangle\langle\mathrm{J}(\vec{k}_{0})|n(\vec{k}_{0}+\delta\vec{k})\rangle$

We name$C_{Jn}=\langle\mathrm{J}(\vec{k}_{0})|n(\vec{k}_{0}+\delta\vec{k})\rangle$, then$|n(\vec{k}_{0}+\delta\vec{k})\rangle=\sum_{J} C_{Jn}|\mathrm{J}(\vec{k}_{0})\rangle$

Assume the Hamiltonian is differentiable, then we have

Write as $\delta H(\delta\vec{k})$, we have:$H(\vec{k}_{0}+\delta\vec{k})=H(\vec{k}_{0})+\delta H(\delta\vec{k})$

Substitute them into the Hamiltonian$H(\vec{k}_{0}+\delta\vec{k})|n(\vec{k}_{0}+\delta\vec{k})\rangle=\omega_{n}(\vec{k}_{0}+\delta\vec{k})|n(\vec{k}_{0}+\delta\vec{k})\rangle$

We have:$(H(\vec{k}_{0})+\delta H(\delta\vec{k}))\sum_{J} C_{Jn}|\mathrm{J}(\vec{k}_{0})\rangle=\omega_{n}(\vec{k}_{0}+\delta\vec{k})\sum_{J} C_{Jn}|\mathrm{J}(\vec{k}_{0})\rangle$

Organize the formula, we end up with a new Hamiltonian:

$\begin{matrix} \hat{H}C_{n}=\omega_{n}(\vec{k}_{0}+\delta\vec{k})C_{n} \\ \hat{H}=\sum_{I,J} [\langle\mathrm{I}(\vec{k}_{0})|\delta H(\delta\vec{k})|\mathrm{J}(\vec{k}_{0})\rangle+\omega_{J}(\vec{k}_{0})\delta_{IJ}] \end{matrix}$ (9)

Extend each components of the Himation in main text equation (6)

$$H= \left[\begin{matrix} & & & & ck_{z} & -ck_{y} & & -i\omega_{p} & & \\ & & & -ck_{z} & & ck_{x} & & & -i\omega_{p} & \\ & & & ck_{y} & -ck_{x} & & & & & -i\omega_{p} \\ & -ck_{z} & ck_{y} & & & & & & & \\ ck_{z} & & -ck_{x} & & & & & & & \\ -ck_{y} & ck_{x} & & & & & & & & \\ & & & & & & & \omega_{p}\kappa k_{x}/k_{p} & \omega_{p}\kappa k_{y}/k_{p} & \omega_{p}\kappa k_{z}/k_{p} \\ i\omega_{p} & & & & & & \omega_{p}\kappa k_{x}/k_{p} & & \frac{i\omega_{c}}{c} & \\ & i\omega_{p} & & & & & \omega_{p}\kappa k_{y}/k_{p} & -\frac{i\omega_{c}}{c} & & \\ & & i\omega_{p} & & & & \omega_{p}\kappa k_{z}/k_{p} & & & \end{matrix}\right] \left(10\right)$$

As above these degeneracies points are band crossing between the longitude mode and the right-circled wave, which eigenstates respectively as below:

$$\begin{matrix} |I\rangle=\left(\begin{matrix} \begin{matrix} \begin{matrix} \begin{matrix} E_{x} & E_{y} \end{matrix} & 0 & H_{x} \end{matrix} & H_{y} & 0 & 0 \end{matrix} & j_{x} & j_{y} & 0 \end{matrix}\right)^{T} 11(a) \\ |J\rangle=\left(\begin{matrix} \begin{matrix} \begin{matrix} \begin{matrix} 0 & 0 \end{matrix} & E_{z} & 0 \end{matrix} & 0 & 0 & n_{1} \end{matrix} & 0 & 0 & j_{z} \end{matrix}\right)^{T} 11(b) \end{matrix}$$

Substitutes (10) and 11(a), (b) into equation (9) obtains,

$$H_{1}=\left[\begin{matrix} \frac{A+N}{2}k_{z} & M\left(k_{x}+ik_{y}\right) \\ M\left(k_{x}-ik_{y}\right) & -\frac{A+N}{2}k_{z} \end{matrix}\right]+\frac{A-N}{2}Ik_{z}$$

Assuming $\kappa$ is close to zero, N can be expressed as:

$$N=\frac{\omega_{p}^{2}\sqrt{\omega_{c}}}{2(\omega_{p}+\omega_{c})\sqrt{\omega_{c}-\omega_{p}}}\kappa^{2}$$

Which is second order in $\kappa$

$$\begin{matrix} A=\frac{\sqrt{\varepsilon_{12}}}{2(\varepsilon_{12}^{2}-\varepsilon_{12}+2)} \\ M=\frac{\sqrt{\varepsilon_{12}}}{2\sqrt{\varepsilon_{12}^{2}-\varepsilon_{12}+2}} \end{matrix}$$

And $\varepsilon_{12}=\varepsilon_{1}+\varepsilon_{2}$is sum of the diagonal and off-diagonal of the dielectric matrix evaluated at the Weyl’s frequency:

$$\omega_{weyl}=\omega_{p}+\frac{\omega_{p}^{2}\omega_{c}}{2(\omega_{c}^{2}-\omega_{p}^{2})}\kappa^{2}$$

And the kz position of the Weyl point:

$$k_{z}^{weyl}=\frac{\omega_{p}}{c}\sqrt{\frac{\omega_{c}}{\omega_{c}-\omega_{p}}}(1+\frac{\omega_{p}^{2}\omega_{c}}{2(\omega_{c}^{2}-\omega_{p}^{2})}\kappa^{2})$$

, which are also second order in $\kappa$ when $\kappa$ is small.

Note 4

*Fermi arc in the dissipating plasmas*

As the type two Weyl points exists in the warm magnetohydrodynamics plasmas proved above, the dissipating non-hermitian effective Hamiltonian shows,

$$h=v_{0}k_{z}\sigma_{0}+v_{1}k_{x}\sigma_{x}+v_{2}k_{y}\sigma_{y}+v_{3}k_{z}\sigma_{z}+ir\sigma_{z} \left(13\right)$$

By solving the 2-by-2 matrix, we could obtain the spectrum dispersion along the x-axis direction, which is also named the bulk fermi disk,

$$E^{2}=\left(k_{x}v_{1}\right)^{2}-r^{2} \left|k_{x}\right|\leq\frac{r}{v_{1}} \left(14\right)$$

To simply the equation, here we perform the unitary transformations:

$$H=\left(U_{y}U_{Z}\right)h\left(U_{y}U_{Z}\right)^{+} \left(15\right)$$

Where

$$U_{y}=e^{i\frac{\pi}{4}\sigma_{y}}, U_{z}=e^{i\frac{\pi}{4}\sigma_{z}} \left(16\right)$$

And

$$\begin{matrix} \left(U_{y}U_{Z}\right)\sigma_{z}\left(U_{y}U_{Z}\right)^{+}=-\sigma_{x} \left(U_{y}U_{Z}\right)\sigma_{x}\left(U_{y}U_{Z}\right)^{+}=-\sigma_{y} \left(17a\right) \\ \left(U_{y}U_{Z}\right)\sigma_{y}\left(U_{y}U_{Z}\right)^{+}=\sigma_{z} \left(U_{y}U_{Z}\right)\sigma_{0}\left(U_{y}U_{Z}\right)^{+}=\sigma_{0} \left(17b\right) \end{matrix}$$

Substitutethese equations,

$$H=v_{0}k_{z}\sigma_{0}-v_{1}k_{x}\sigma_{y}+v_{2}k_{y}\sigma_{z}-v_{3}k_{z}\sigma_{x}-ir\sigma_{x} \left(18\right)$$

And the eigenstate equation can be written as

$$\left[\begin{matrix} v_{0}k_{z}+v_{2}k_{y}-E & -v_{3}k_{z}+iv_{1}k_{x}-ir \\ -v_{3}k_{z}-iv_{1}k_{x}-ir & v_{0}k_{z}-v_{2}k_{y}-E \end{matrix}\right] |\psi\rangle=0 \left(20\right)$$

For the surface states which localized at the boundary,$k_{y}=k_{y1}+ik_{y2}$. Hashimoto et. al [1] have demonstrated that a generic boundary condition,

Combined with the boundary condition, the localized modes can be written as$e^{-ay}\left[\begin{matrix} 1 \\ e^{i\theta} \end{matrix}\right]$,

Substitute equation (20) to the eigenstate equation which gives,

$$\begin{matrix} \left[\begin{matrix} v_{0}k_{z}+v_{2}\left(k_{y1}+ik_{y2} \right)-E & -v_{3}k_{z}+iv_{1}k_{x}-ir \\ -v_{3}k_{z}-iv_{1}k_{x}-ir & v_{0}k_{z}-v_{2}\left(k_{y1}+ik_{y2} \right)-E \end{matrix}\right]e^{-ay}\left[\begin{matrix} 1 \\ e^{i\theta} \end{matrix}\right]=0 \left(22\right) \\ \end{matrix}$$

For the real and imaginary parts of the equation above, we can obtain,

$$\begin{matrix} E_{1}= \frac{\left(v_{0}k_{z}+ \sin\theta\left(- 2v_{1}k_{x}+v_{0}k_{z}\sin\theta\right)-\cos\theta\left(2v_{3}k_{z}-v_{0}k_{z}\cos\theta\right)\right)}{2} \left(23a\right) \\ E_{2}=-r\cos\theta\left(23b\right) \\ k_{y1}=-v_{0}k_{z}-r\sin\theta-v_{3}k_{z}\cos\theta-\frac{2v_{1}k_{x}\sin\theta-v_{0}k_{z}+r\sin\theta+v_{3}k_{z}\cos\theta}{2v_{2}} \left(23c\right) \\ k_{y2}=r\cos\theta-v_{1}k_{x}\cos\theta+\frac{2v_{3}k_{z}\sin\theta-r\cos\theta-v_{1}k\cos\theta}{2v_{2}} \left(23d\right) \end{matrix}$$

We have shown the different boundary parameter $\theta$ for the fermi arc energy dispersion In the supplementary figure 8.

To simply the beginning or the ending of the fermi arc, we can also take a very simple case, for the bulk states at the bulk fermi arc, where $E=i\varepsilon$, $k_{z}=0$

$$\left[\begin{matrix} v_{2}k_{y}-i\varepsilon& +iv_{1}k_{x}-ir \\ -iv_{1}k_{x}-ir & -v_{2}k_{y}-i\varepsilon\end{matrix}\right]\left[\begin{matrix} a \\ b \end{matrix}\right]=0 \left(25\right)$$

We could obtain the form of the eigenstates

Combing with $r^{2}=\left(v_{2}k_{y}\right)^{2}+\varepsilon^{2}+\left(v_{1}k_{x}\right)^{2}$from equation (20), the only corresponding solution is $k_{x}=0$. This is also corresponding with equation (23). So, the dissipatingfermi arc is not connected with the Els, while in the middle of the bulk fermi disk under similar boundary condition to Hermitian Hamiltonians.

Note 5

*Berry curvatures of non-hermitian Hamilton*

In the dissipative plasma systems, these degeneracies are exceptional loops that are defective, unitarily similar to Jordan matrix and cannot span the full Hilbert space. Although the right eigenstate and the left eigenstate share same eigenvalue, they are not the same as the matrix $\hat{H}^{+}\neq\hat{H}$. Right eigenstate of $\hat{H}$and left eigenstate of the adjointoperator$\hat{H}^{+}$satisfy, and.These two eigenstates are biorthogonal to each other , which$\alpha,\beta=L/R$.Four different gauge invariant Berry curvatures can be formed,

H.T. Sen et. al[1] have proved that four corresponding Chern numbers share same values which depicts that the topological invariant could be presented by just a single Chern number. Thereupon we study the$B_{n}^{RR}\left(\vec{k}\right)$by the method of wisloop to performance the flux in a numeral calculations method:

Where the subscripts 1 to 4 denote the eigen states on a square infinitesimally close to $\vec{k}$.

We also derive the non-Hermitian berry curvatures by the effective Hamilton. First, let’s look at the type IInon-Hermitian Fermi disk Hamiltonian, to simply, here we assume $v_{0,1,2}=0$and $v_{3}=0$,

$$H=(k_{z}+i\gamma)\sigma_{z}+k_{x}\sigma_{x}+k_{y}\sigma_{y}+vk_{z}I$$

Same as the Hermitian case for type II Hamiltonian, the transformation to new energy and momentum coordinate can reduce it into a type I form without the unit matrix. $E'=E-vk_{z}$,Then the Hamiltonian reads:

$$H=(k_{z}+i\gamma)\sigma_{z}+k_{x}\sigma_{x}+k_{y}\sigma_{y}$$

Whose eigen-energy reads:$E=\pm\sqrt{(k_{z}+i\gamma)^{2}+r^{2}}$ , $re^{i\theta}=k_{x}+ik_{y}$

Due to the non-hermicity, we define the left and right eigen states as:

and

Naturally we have

and

Notice that $\eta=\sqrt{(k_{z}+i\gamma+E)^{2}+r^{2}}$ to satisfy the normalization condition

First, we look at the Berry connections, there are genuinely four ways to define the berry connections, which are:$A_{\theta}^{IJ}=\langle I|\partial_{\theta}|J\rangle$,

The explicit form of the four Berry connections are:

$$\left[\begin{matrix} A_{\theta}^{\phi\phi} & A_{\theta}^{\phi\varphi} \\ A_{\theta}^{\varphi\phi} & A_{\theta}^{\varphi\varphi} \end{matrix}\right]=\left[\begin{matrix} -\frac{r^{2}}{\left|\eta\right|^{2}} & -\mathrm{Re}(\frac{r^{2}}{\eta^{2}}) \\ -\mathrm{Re}(\frac{r^{2}}{\eta^{*2}}) & -\frac{r^{2}}{\left|\eta\right|^{2}} \end{matrix}\right]$$

Note that for the off-diagonal terms, the Berry connection is not pure real, and we just use the real part, standing for the geometrical phase.

For all four cases, it’s intuitive to show that the Chern number are equivalent by computing

$$C^{IJ}=\lim_{r\to0} A_{\theta}^{IJ}-\lim_{r\to\infty} A_{\theta}^{IJ}$$

For all cases, we can conclude

For energy band$E_{up}=\sqrt{(k_{z}+i\gamma)^{2}+r^{2}}$, $C=-\pi$

For energy band$E_{low}=-\sqrt{(k_{z}+i\gamma)^{2}+r^{2}}$, $C=\pi$

Hence when we look at the upper band expressed by $E=sign(k_{z})\sqrt{(k_{z}+i\gamma)^{2}+r^{2}}$

The total Chern number emitted by the Fermi disk is $2\pi$.We comment that the local Berry curvatures have the relations:$A_{\theta}^{\phi\varphi}=A_{\theta}^{\varphi\phi}\neq A_{\theta}^{\phi\phi}=A_{\theta}^{\varphi\varphi}$.Here also we give expression of the asymptotic Berry curvature of$A_{\theta}^{\phi\varphi}$. We consider the case the integrated Berry curvature on small disk in proximity to the Fermi disk but whose radiussatisfy$0<k_{z}\ll r\ll\gamma$, easily we have,

$$\begin{matrix} \int_{0}^{r} \Omega_{z}(t,k_{z},\gamma)tdt=2\pi\mathrm{Re}(\frac{r^{2}}{(k_{z}+i\gamma+E)^{2}+r^{2}}) \\ \frac{r^{2}}{(k_{z}+i\gamma+\sqrt{(k_{z}+i\gamma)^{2}+r^{2}})^{2}+r^{2}}=\frac{r^{2}}{(k_{z}+i\gamma+\sqrt{k_{z}^{2}+2i\gamma k_{z}-\gamma^{2}+r^{2}})^{2}+r^{2}} \\ \approx\frac{r^{2}}{(k_{z}+i\gamma+\sqrt{2i\gamma k_{z}-\gamma^{2}})^{2}+r^{2}}=\frac{r^{2}}{(k_{z}+i\gamma+i\gamma(1-\frac{i}{\gamma}k_{z}))^{2}+r^{2}} \\ =\frac{r^{2}}{(2k_{z}+2i\gamma)^{2}+r^{2}}=\frac{r^{2}}{4k_{z}^{2}-4\gamma^{2}+8i\gamma k_{z}+r^{2}} \\ \approx\frac{r^{2}}{-4\gamma^{2}+8i\gamma k_{z}}=-\frac{1}{4\gamma}\frac{r^{2}}{\gamma-2ik_{z}} \\ \mathrm{Re}(-\frac{1}{4\gamma}\frac{r^{2}}{\gamma-2ik_{z}})=-\frac{r^{2}}{4(\gamma^{2}+4k_{z}^{2})} \end{matrix}$$

Differentiating both side, we have

$$\Omega_{z}(r,k_{z},\gamma)=-\frac{1}{(\gamma^{2}+4k_{z}^{2})}$$

The conclusion is that we can see that when $k_{z}\ll r\ll\gamma$the Berry curvature is uniform on r.

Following similar calculations $k_{z}<0$,

$$\Omega_{z}(r,k_{z},\gamma)=\frac{1}{(\gamma^{2}+4k_{z}^{2})}$$

Hence consider a closed sphere that encloses the Bulk Fermi disk, the integrated Berry curvature is always nonzero.

Next let’s look at another approximation near to the ring, first we do another basis transformation to make the Hamiltonian:

$$H=\left[\begin{matrix} k_{x} & k_{z}+i\gamma+ik_{y} \\ k_{z}+i\gamma-ik_{y} & -k_{x} \end{matrix}\right]$$

Now we let$k_{z}+ik_{y}=i\gamma+re^{i\theta}$, then the Hamiltoniantransforms as

$$H=\left[\begin{matrix} k_{x} & 2i\gamma+re^{i\theta} \\ re^{-i\theta} & -k_{x} \end{matrix}\right]$$

And the eigenvalue $E^{2}=k_{x}^{2}+r^{2}+2i\gamma re^{-i\theta}$, $E\approx\sqrt{2i\gamma r}e^{-i\frac{\theta}{2}}=\sqrt{2\gamma r}e^{i\frac{\pi}{4}}e^{-i\frac{\theta}{2}}$. The eigen states we are looking for are:

and

Where $\eta^{2}=2rEe^{-i\theta}=\sqrt{8i\gamma r^{3}}e^{-i\frac{3\theta}{2}}$,

Calculating the Berry connections, we have$A_{\theta}^{\phi\varphi}=i\frac{k_{x}}{\sqrt{8\gamma r}}e^{i(\frac{\theta}{2}-\frac{\pi}{4})}$,$A_{k_{x}}^{\phi\varphi}=\frac{1}{\sqrt{8\gamma r}}e^{i(\frac{\theta}{2}-\frac{\pi}{4})}$.

And the Berry curvature is $\Omega_{k_{x}\theta}=imag\left(\partial_{k_{x}}A_{\theta}^{\phi\varphi}-\partial_{\theta}A_{k_{x}}^{\phi\varphi}\right)=\frac{1}{4\sqrt{2\gamma r}}\cos(\frac{\theta}{2}-\frac{\pi}{4})$. Note that the inverse square roots term means the Berry curvature grows very slowly towards the exceptional ring.Since $E\approx\sqrt{2i\gamma r}e^{-i\frac{\theta}{2}}=\sqrt{2\gamma r}\{\cos(\frac{\theta}{2}-\frac{\pi}{4})-i\sin(\frac{\theta}{2}-\frac{\pi}{4})\}$, By choosing $\mathrm{Re}(E)>0$ or $\mathrm{Re}(E)<0$will have opposite Berry curvatures.

Here we integrate the Berry curvature on a torus:

$$C=2\pi\gamma\int_{\alpha}^{\alpha+2\pi} \Omega_{k_{x}\theta}rd\theta$$

Substitute the Berry curvature, $C=\frac{\pi}{2}\sqrt{\frac{\gamma r}{2}}\int_{\alpha}^{\alpha+2\pi} \cos(\frac{\theta}{2}-\frac{\pi}{4})d\theta$.Since here the term $\cos(\frac{\theta}{2}-\frac{\pi}{4})$ is essentially representing the real part of the spectrum, which should be kept always >0 or <0, which mean $\alpha=-\frac{\pi}{2}$ or $\alpha=\frac{3\pi}{2}$, Thus $C=\pm\pi\sqrt{\frac{\gamma r}{2}}$

This is a spectacular result, meaning that if we integrate Berry curvature on a negligibly small torus, the Chern number is essentially zero. This means that the Berry curvature is essentially spread on the Fermi disk, and the Berry curvature emitted only by the exceptional loop is essentially zero.

Note 6

*Topological dissipating surface states of the exceptional loops.*

To solve for the surface states, the Magnetohydrodynamics (MHD) plasma is put in a sharp boundary configuration against vacuum, which is a scenario found in magnetic confinement plasmas such as Tokamak configurations. In such case, additional boundary condition dictating the current density to diminish in the surface normal direction should be incorporated into the conventional electromagnetic wave continuity conditions [2]. Due to the continuity of bulk displacement current normal to the interface, this additional boundary condition is equivalent to continuity of Ex across the interface.Surface wave dispersions near the outer exceptional loop is given in FIG. S4. Numerical results with $k_{z}$ smaller, equal and larger than the exceptional loop are given in FIG. S4b-5d and 5h-5g. Note that while the real part of the eigen frequency is outside the real part band gap, the imaginary part could be found in the bulk spectrum’s imaginary frequency. As is shown in FIG. S4, even the real part of the surface states are within the real band gap. The corresponding imaginary part is with the spectrum, meaning the surface states are not localized to the boundary, but a “waveguide” mode to our solved problem, and whose eigen energy varies with the waveguide’s geometry.

Note 7

*Higher order momentum terms on nodal disks*

Higher order terms could have various effects on the nodal disks. First, the disks could have large curvature, as is the case for large viscosity effect. Second, the disk could exist not at the same frequency. The two effects could be captured by quadratic momentum terms at different matrix entries

$$H=(k_{z}+i\gamma+\alpha k_{r}^{2})\sigma_{z}+k_{x}\sigma_{x}+k_{y}\sigma_{y}+\beta k_{r}^{2}$$

Where $k_{r}^{2}=k_{x}^{2}+k_{y}^{2}$. When $\alpha\neq0, \beta=0$, the nodal disk can be described by: $E=\pm i\gamma, k_{z}=-\alpha k_{r}^{2}$, as is shown by the solid black line in Figure. S10 (a). When $\alpha=0, \beta\neq0$, $E=\pm i\gamma+k_{r}^{2}, k_{z}=0$, as is shown in Figure. S10 (b).


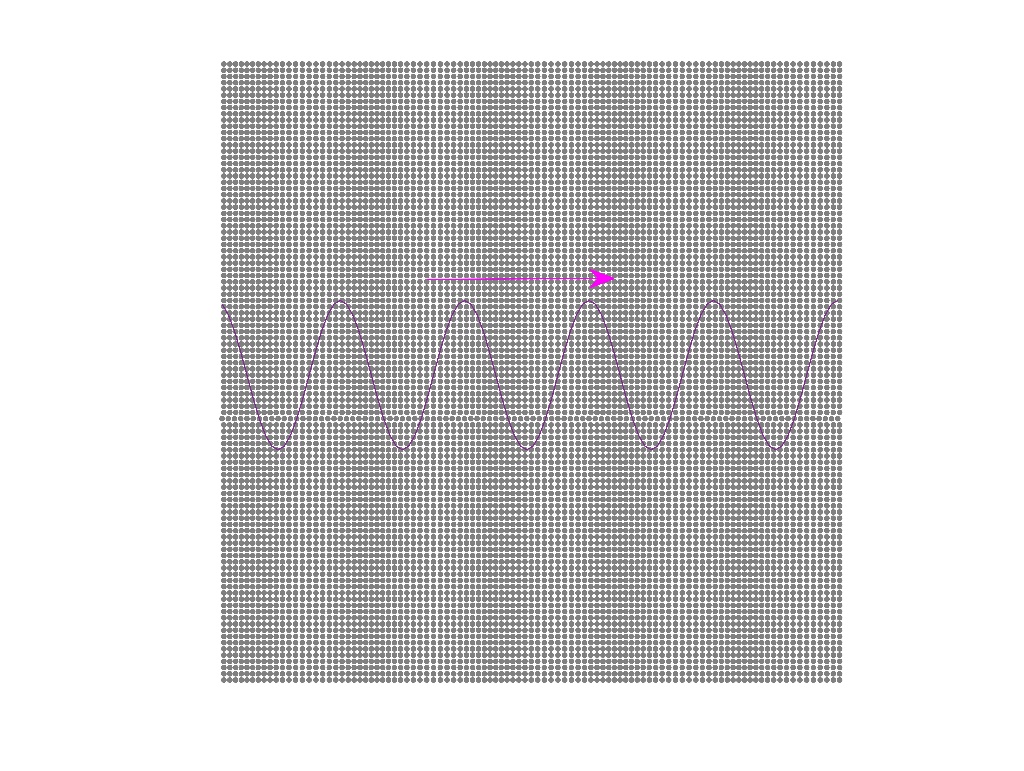


FIG.S1 Langmuir waves. Thermal press acts as restoring force which push the rapid oscillations of the [electron density](https://en.wikipedia.org/wiki/Electron_density) longitude propagated


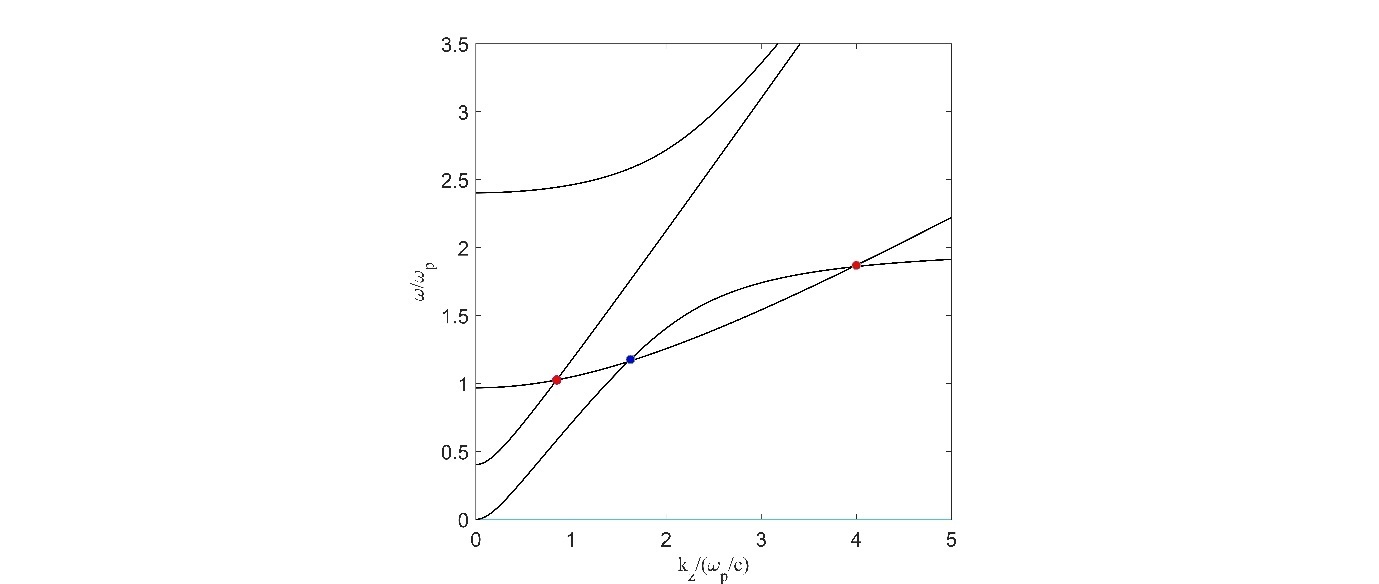


FIG.S2 Existence of Weyl points in magnetized warm ideal plasma. Dispersion of the absolute value of eigen frequency $\omega$ along the $k_{z}$ axis with $\omega_{c}=2, \kappa=0.4$. These dependency points are highlighted by solid color spots which the red one for +1 and the blue one for -1.


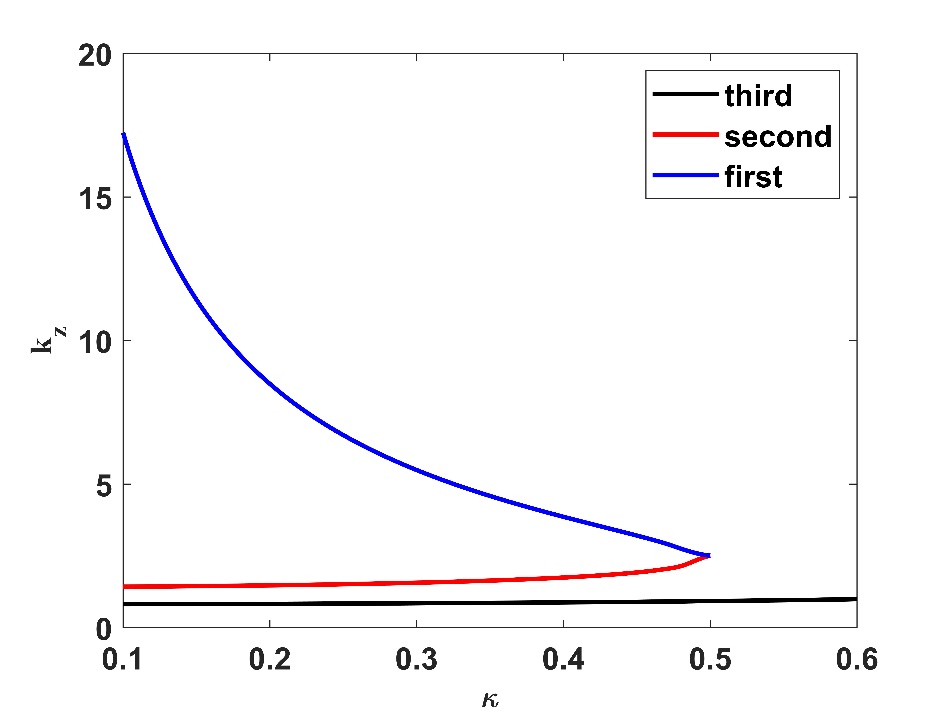


FIG.S3 Positions of the three pairs of Weyl points. The black, red and blue solid line represents the inner the middle and the outer Weyl points. The merge of the red and blue line demonstrates the outer two Wely points getting closer and annihilation.


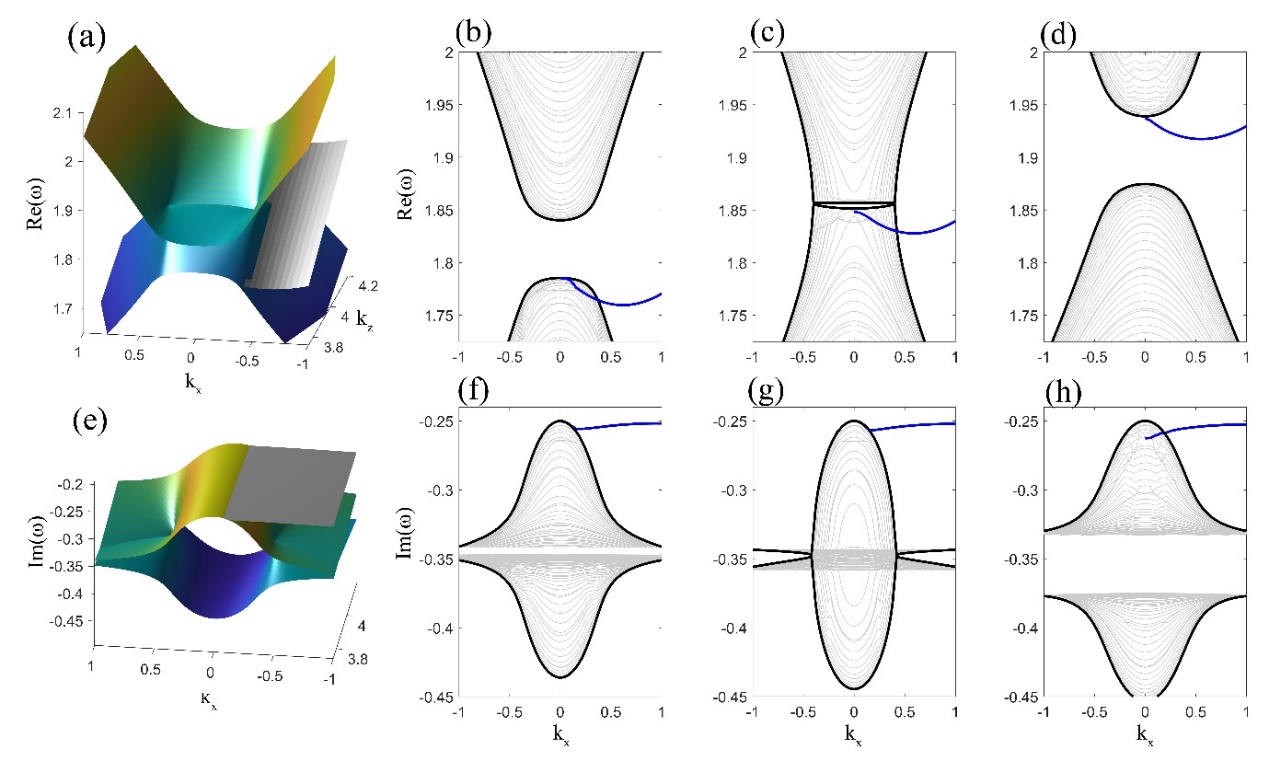


FIG.S4. Surface states between the magnetohydrodynamic plasma and vacuum around the exceptional loops. (a) Real part of the surface wave and the bulk wave dispersions around the exceptional loop. (b)-(d) numerically simulated surface states (blue), bulk states (grey) and the bulk states calculated from the Hamiltonian formalism (black) at different $k_{z}$. (e)-(h) Imaginary part of the surfaces.


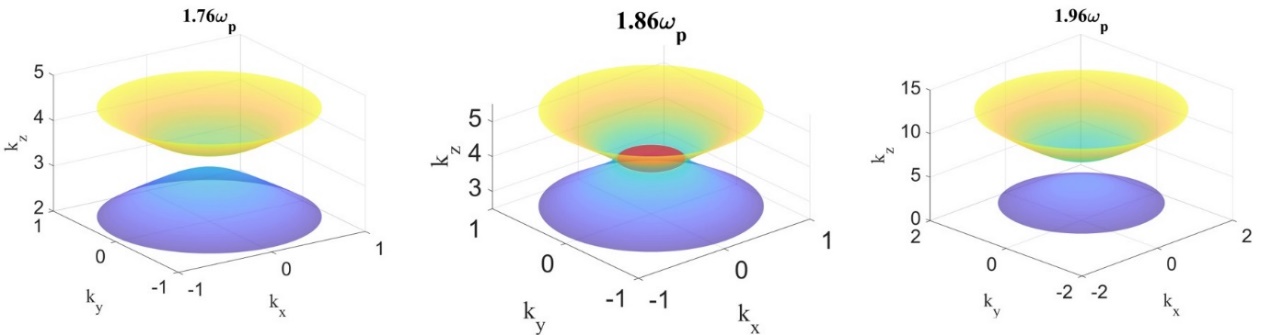


FIG.S5. Three-dimensional equal frequency contour at shifted frequency which are larger (1.96 $\omega_{p}$), equals (1.86 $\omega_{p}$) and smaller (1.76 $\omega_{p}$) bulk fermi disk frequency. The red degeneracy disk is the bulk fermi disk.


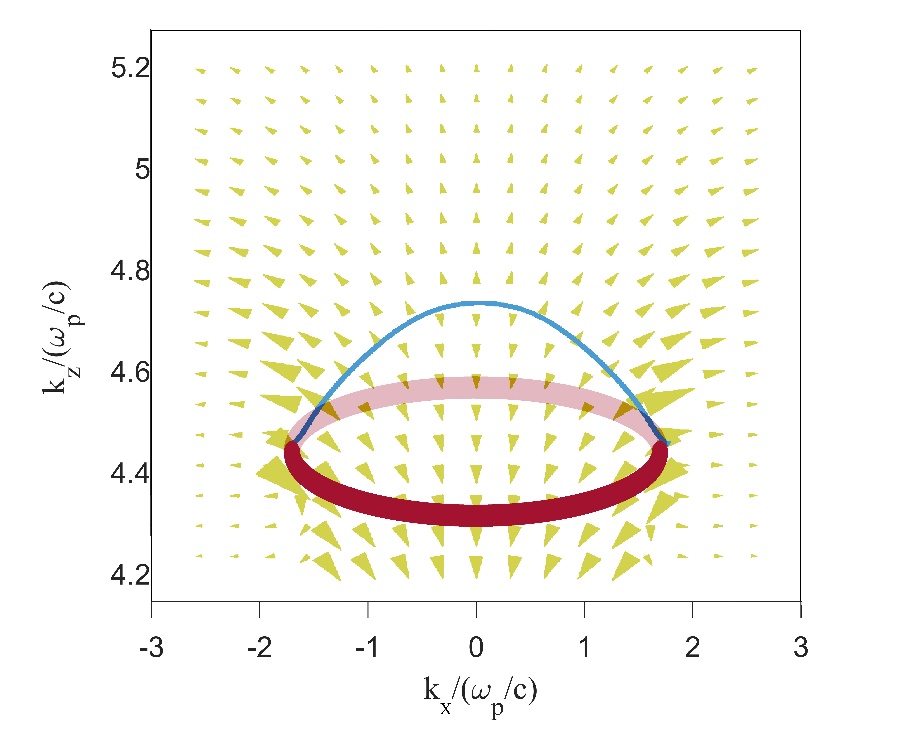


FIG.S6. Berry curvature for the upper band of viscosity warm magnetic plasma in the $k_{x}-k_{z}$ plane. The red rings are the Els in $k_{x}-k_{y}$ plane and the blue solid line is the bulk fermi arc in $k_{x}-k_{z}$ plane. Scales of the arrow represent the intensity of Berry curvatures and orientation indicates the direction. The arrows show that the EL and the degeneracy line are pure source of Berry curvature. The real degenerate line in viscosity warm magnetic plasma is heavily curved comparing to the collision warm magnetic plasma.


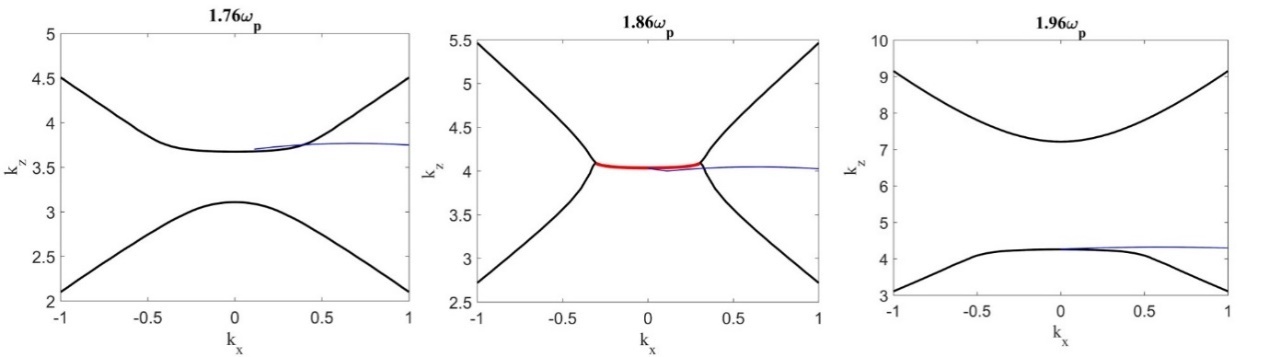


FIG.S7. dispersion of the fermi arc between the dissipating plasma and vacuum at the frequency larger (1.96 $\omega_{p}$), equals (1.86 $\omega_{p}$) and smaller (1.76 $\omega_{p}$) bulk fermi disk frequency. The red degeneracy line is the projection of the bulk fermi disk.


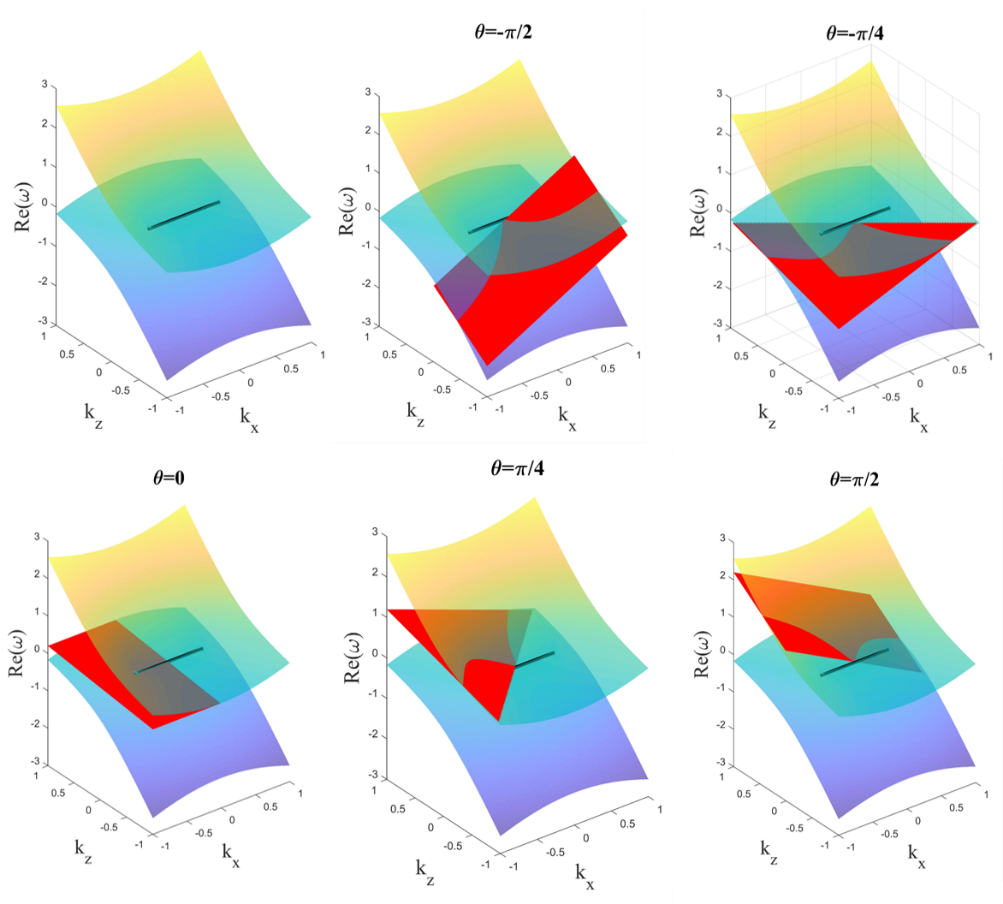


FIG.S8. energy dispersion for the surface mode and the bulk mode respectively for the boundary condition parameter for $\theta=\frac{pi}{2},\frac{pi}{4}, 0, -\frac{pi}{4}, -\frac{pi}{2}$. The red plane are the surface modes.


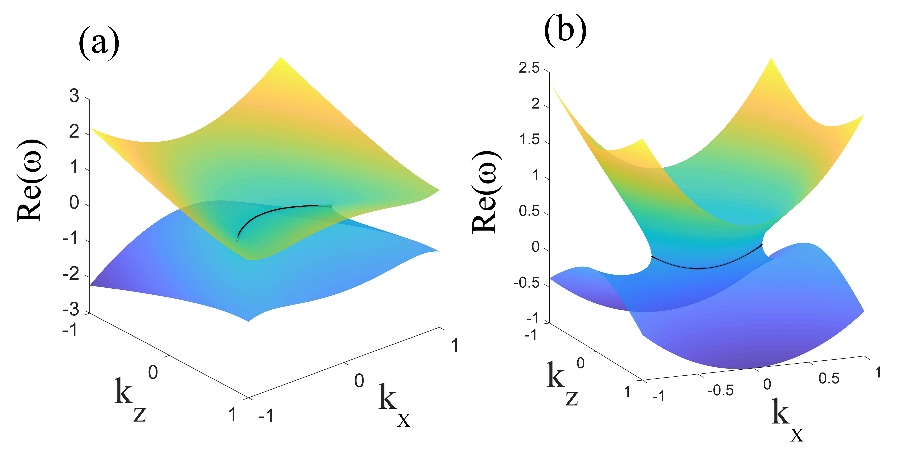


FIG.S9. Dispersion and the nodal disks under different quadratic terms. When $\alpha\neq0, \beta=0$, the nodal disk can be described by: $E=\pm i\gamma, k_{z}=-\alpha k_{r}^{2}$. When $\alpha=0, \beta\neq0$, $E=\pm i\gamma+k_{r}^{2}, k_{z}=0$.

[1] K. Hashimoto, T. Kimura and X. Wu, Prog. Theor. Exp. Phys. 053I01(2017).

[2] H. T. Shen, B. Zhen and L. Fu, Phys. Rev. Lett. 120, 146402 (2018).

[3] H. J. Lee and S. H. Cho, J. Plasma Phys, 53, 409 (1997).
